# Glycogenosome accumulation in the arrector pili muscle in Pompe disease

**DOI:** 10.1186/1750-1172-9-17

**Published:** 2014-02-05

**Authors:** Istvan Katona, Joachim Weis, Frank Hanisch

**Affiliations:** 1 Institute of Neuropathology, RWTH Aachen University and JARA Brain Translational Medicine, Pauwelsstr. 30, 52074 Aachen, Germany; 2 Department of Neurology, Martin Luther University Halle-Wittenberg, Ernst-Grube-Str. 40, 06120 Halle (Saale), Germany

**Keywords:** Skin biopsy, Pompe disease, Glycogenosis type II, Arrector pili muscle, Glycogenosome, Autophagy

## Abstract

**Background:**

Glycogenosis type II or Pompe disease is an autosomal-recessive lysosomal storage disease due to mutations in the gene encoding acid alpha-glucosidase (GAA), an enzyme required for lysosomal glycogen degradation. The disease predominantly affects the skeletal and respiratory muscles but there is growing evidence of the involvement of smooth muscle cells in blood vessel walls, suggesting a multi-system disorder. Moreover, the failure of autophagy in Pompe disease could contribute to muscular atrophy and disease progression and is thought to compromise the efficacy of enzyme replacement therapy (ERT).

**Methods:**

We investigated the light microscopical and ultrastructural pathology of the arrector pili muscle from punch skin biopsies from the calf of 6 adult Pompe disease patients and 6 age and gender matched healthy controls. Two patients had a follow-up biopsy after 19 and 20 month of ERT.

**Results:**

The electron microscopic investigation of patient biopsies revealed the widespread occurrence of glycogenosomes, membrane bound accumulations of granular glycogen, associated with autophagic vacuoles. In the controls we detected only muscle cells with non-membrane bound forms of glycogen. These morphological changes in smooth muscle cells are similar to those seen in skeletal muscle and smooth muscle cells of arterioles of Pompe patients. Furthermore, two patients with pre- and post-ERT skin biopsies showed a decrease in the number of cells with extensive autophagy after treatment.

**Conclusions:**

Electron microscopic examination of the arrector pili muscles appears to be a surrogate marker for the involvement of smooth muscles reflecting disease severity. These findings suggest that the standardized and widely used skin biopsy could offer a minimally invasive way to screen for smooth muscle involvement and warrant further studies in larger cohorts of patients.

## Introduction

Glycogenosis type II or Pompe disease (MIM #232300), is an autosomal recessive lysosomal storage disease due to mutations in the gene encoding acid α-glucosidase (GAA), an enzyme required for glycogen degradation in lysosomes. Phenotypes range from a severe neonatal onset form with cardiac involvement to a milder adult onset form, which is characterized by skeletal and respiratory muscle weakness [1-3]. Intravenous enzyme replacement therapy (ERT) with recombinant α-glucosidase has been available and is approved for children and adults since 2007 [4,5].

The use of skeletal muscle biopsies as an early diagnostic tool is an invasive procedure and can lead to false negative results [1-3,6]. A blood-based GAA enzyme activity assay is currently recommended to screen for GAA deficiency and is able to confirm or exclude Pompe disease in all age groups. The diagnosis should be confirmed either by a second GAA enzyme activity assay or GAA sequencing [1-3,6].

There is growing evidence for an involvement of smooth muscle fibers indicating a multi-system disorder [5,7-10]. Pathological glycogen accumulation has been found in smooth muscles cells of arteries, veins, and bronchioles [5,7,11]. ERT has led to the reduction of glycogen aggregates in the smooth muscle cells of arteries and veins in a knockout mouse model of Pompe disease and patients with infantile Pompe disease [5,7].

Skin punch biopsy is an established and standardized procedure to analyse the arrector pili muscle, composed of smooth muscle cells [12-14]. In the present study we analysed the arrector pili muscle in skin punch biopsies from adult patients with Pompe disease prior to and during ERT and compared the glycogen accumulation with normal controls.

## Methods

### Patients, controls, biopsy material

We examined 6 adult patients (2 males, 4 females; mean age 48,5 years) with biochemically and genetically confirmed Pompe disease. At the time point of biopsy 2 of the patients had been receiving ERT and 4 had not yet started ERT. In 2 of the latter patients, a second biopsy was performed after commencing ERT. All patients gave their informed consent. The study was approved by the local ethics committee of Martin-Luther-University Halle (Saale). Skin biopsies from 6 healthy individuals obtained from the diagnostic skin biopsy archive of the Institute of Neuropathology, RWTH Aachen University, were used as controls.

A 3 mm skin punch biopsy was taken from the standard location of 10 cm proximally of the lateral malleus [14] to screen for glycogen deposits and vacuoles in smooth muscle cells of the arrector pili muscles by light and electron microscopy. Archived diagnostic skeletal muscle biopsies obtained from six adult patients (2 male; 4 female; mean age 43.7 years) with genetically confirmed, untreated Pompe disease were also analysed by light and electron microscopy. These were used to compare the ultrastructural morphological changes in skeletal and arrector pili muscle with special focus on glycogen accumulation and vacuolisation within skeletal muscle fibers, endothelial cells and smooth muscle cells of the small intramuscular blood vessels. Skeletal muscle biopsies prior to ERT were not available for any of the patients undergoing skin punch biopsy.

### Immunohistochemistry, light and electron microscopy

Cryostat sections of formalin fixed, Tissue-Tek O.C.T. compound (Sakura Finetek Europe, Zoeter Woude, The Netherlands) embedded tissues were stained with hematoxylin and eosin (H&E), periodic acid Schiff stain (PAS) and with anti-p62 (Abcam, mouse monoclonal antibody, visualized with HRP conjugated secondary anti-mouse antibody, Cell Signalling Technology). For electron microscopy, glutaraldehyde-fixed tissue specimens were post-fixed with 1% OsO_4_ in 0.1 M cacodylate buffer containing 50 mM K_3_Fe (CN)_6_ and embedded in epoxy resin. Ultramicrotome cut semithin sections from the resin blocks were stained with toluidine blue and paraphenylene diamine. Ultrathin sections were contrast enhanced with uranyl acetate and lead citrate and examined with a Philips EM 400 T electron microscope and images were captured using a Morada digital camera as previously described [15].

### Quantification of glycogen deposition in smooth muscle cells

**Figure 1:**
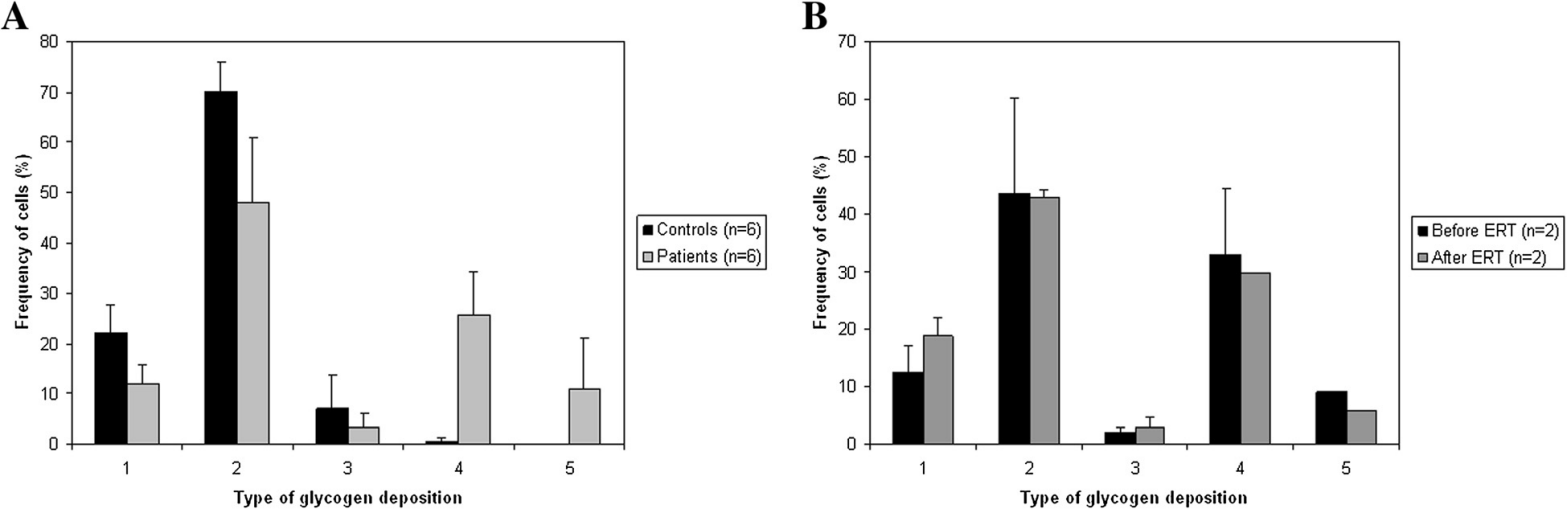
**Quantification of cytoplasmic glycogen content in smooth muscle cells of arrector pili muscle.** The smooth muscle cells were assigned to five categories: 1 - No visible glycogen content; 2 – Scattered glycogen granules; 3 - Large amount of granular glycogen; 4 – Glycogenosomes; 5 – Extensive autophagy, filling a significant part of the cytoplasm. **A**: A large fraction of smooth muscle cells from Pompe patients shows abnormal forms of glycogen accumulation, while the cells in controls contain mainly no or granular glycogen, which is considered as a normal property for this cell type. (Chi square test: p < 0,0001); **B**: The number of cells containing glycogenosomes or presenting extensive autophagy is decreased after ERT in the two patients where a follow up biopsy was available. (Significance could not be determined due to the low subject number).

The glycogen content of arrector pili smooth muscle cells was assessed semiquantitatively using electron micrographs of randomly selected fields on the cross sections of the muscles. The individual smooth muscle cells were then manually examined, counted and assigned to five categories according to their glycogen content (see legend of Figure 1). We counted 849 cells from controls and 1511 cells from Pompe disease patients. The chi-squared test was used to compare the patient and control group regarding the normal and abnormal glycogen accumulations.

## Results

### Clinical data

Clinical characteristics of the adult Pompe patients are given in Table 1. The mean age at examination was 48.5 years. All patients had proximal paresis of arms and legs. No patient was wheelchair-bound or depended on nocturnal non-invasive ventilation. For the two patients (P4 and P5) who had received ERT after the first skin biopsy, the second skin biopsies were obtained 19 and 20 months after ERT, respectively.

**Table 1:** Clinical characteristics and laboratory findings of 6 adult Pompe patients

	**P1**	**P2**^**a**^	**P3**^**a**^	**P4**^**b**^	**P5**^**b**^	**P6**
Sex	Female	Female	Female	Male	Female	Male
Age (year)	22	53	50	47	56	63
Durations of symptoms in years	12	27	24	10	16	11
Duration of ERT in months	24	0	0	0 (19)	0 (20)	12
GAA genotype	c.-45 T > G*; IVS9-1G > C	c.-45 T > G; IVS16 + 102_IVS17 + 31	c.-45 T > G; IVS16 + 102_IVS17 + 31	c.307 T > G; c.1478C > T	c.307 T > G; c.1478C > T	c.-45 T > G: c.925G > A
6 minute walk test (m)	510	130	430	450	420	120
Walking aids	No	Yes	No	No	No	Yes
Walton Gardner Medwin Scale^c^	3	6	2	4	3	6
Slow Vital capacity (%)	60	56	96	91	73	44
α-glucosidase activity						
At pH 3.8	0.59	0.36	1.06	1.17	0.72	1.58
With inhibition (dry blood spot test**)	0.14	0.17	0.39	0.32	0.05	0.18

^a^Siblings.

^b^Siblings.

^c^The Walton-Gardner-Medwin Scale (WGMS) is a 10-item scale to asses the degree of walking disability (0 normal walking, 7 wheelchair bound).

In brackets is the duration of ERT in months at the time of the second biopsy.

ERT enzyme replacement therapy, GAA acid α-glucosidase,

*c.-45 T > G stands for c.-32-13 T > G.

**Normal range of α-glucosidase acitivity at pH 3.8: 1.5 – 10.0 nmol/spot*21 hours; with inhibition: 0.9-7.2 nmol/spot*21 hours.

### Skeletal muscle histology

**Figure 2:**
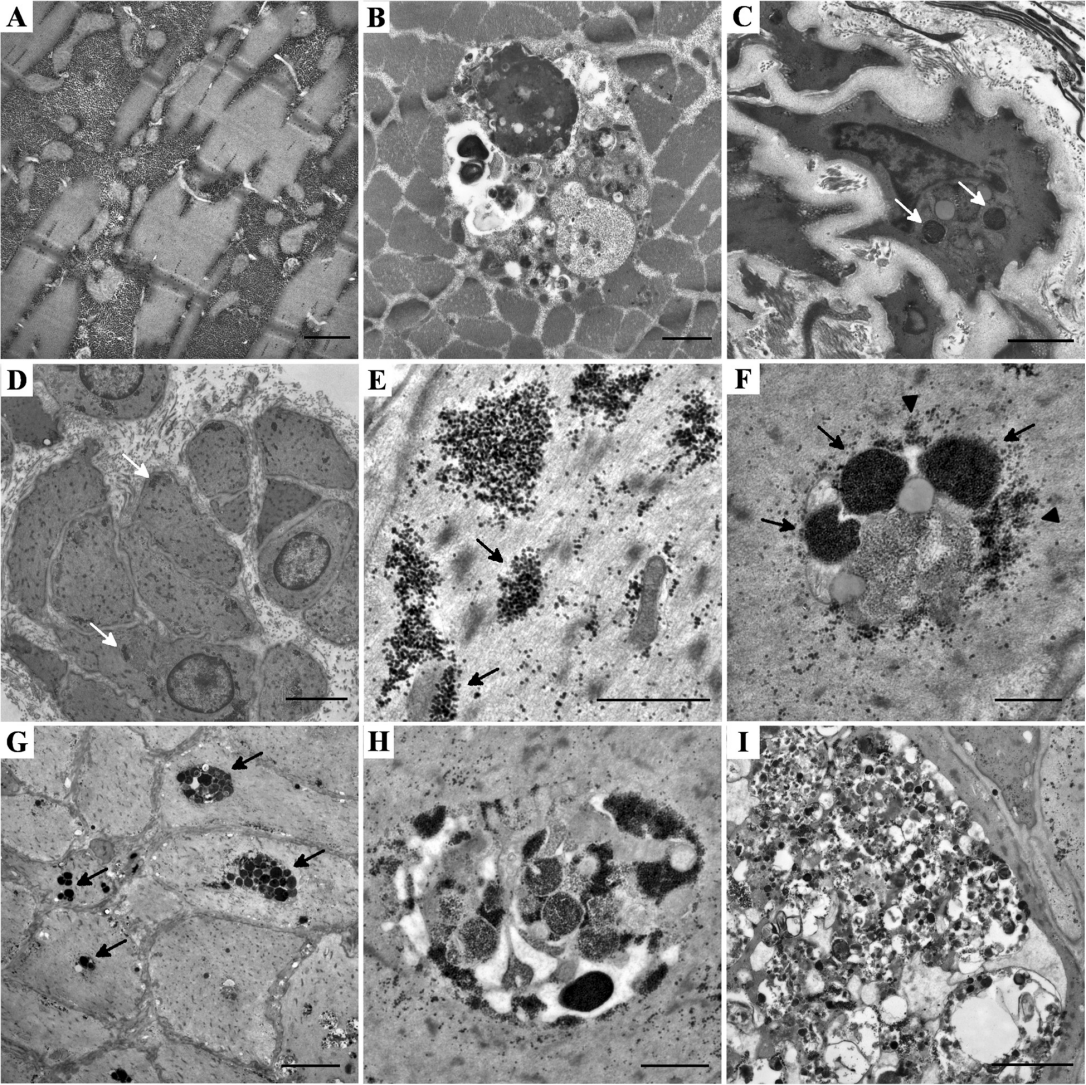
**Electron microscopy of skeletal muscle and arrector pili smooth muscle of Pompe disease patients and controls. A**: Granular glycogen accumulation disrupting the myofibrilar structure in skeletal muscle of a Pompe patient. Scale bar: 0,5 μm. **B**: Intramyofibrilar glycogenosome accumulation with varying density of the glycogen deposits, associated with autophagic vacuoles in the skeletal muscle of a Pompe disease patient. Scale bar: 1 μm. **C**: Large glycogenosomes containing tightly packed glycogen granules (arrows) in a smooth muscle cell of an intramuscular blood vessel from a patients muscle biopsy. Scale bar: 2 μm. **D-E**: Smooth muscle cells of arrector pili muscle from a skin biopsy of a healthy control. Many cells contain granular, non-membrane bound glycogen deposits (arrows) which are frequently associated loosely with the cellular membrane or mitochondria (arrowheads). Scale bar D: 2 μm; E: 1 μm. **F-G**: Glycogen appears in membrane bound (arrows) and also in granular form (arrowhead) in the arrector pili smooth muscle cells of Pompe patients. Scale bar F: 0,5 μm; G: 5 μm. **H**: Smooth muscle cells from arrector pili muscle of Pompe patients contain similar vacuoles that we can observe in skeletal muscle of such patients, containing diverse forms of glycogen and autophagic material. Scale bar: 1 μm. **I**: The glycogenosomes and autophagic vacuoles fill the entire cytoplasm of the arrector pili smooth muscle cells in some cases, severely disrupting their structure. Scale bar: 2 μm.

In PAS and H&E stained paraffin and cryostat sections, in semithin sections stained with toluidine blue and by electron microscopy we observed vast intermyofibrillar and subsarcolemmal glycogen accumulations (Figure 2A) associated with autophagic vacuoles in all Pompe patient-derived muscle biopsies (Figure 2B). Increased and abnormal glycogen accumulations were also found in fibroblasts in the vicinity of endomysial capillaries, in smooth muscle cells of intramuscular arterioles and in small arteries (Figure 2C).

### Skin biopsy

**Figure 3:**
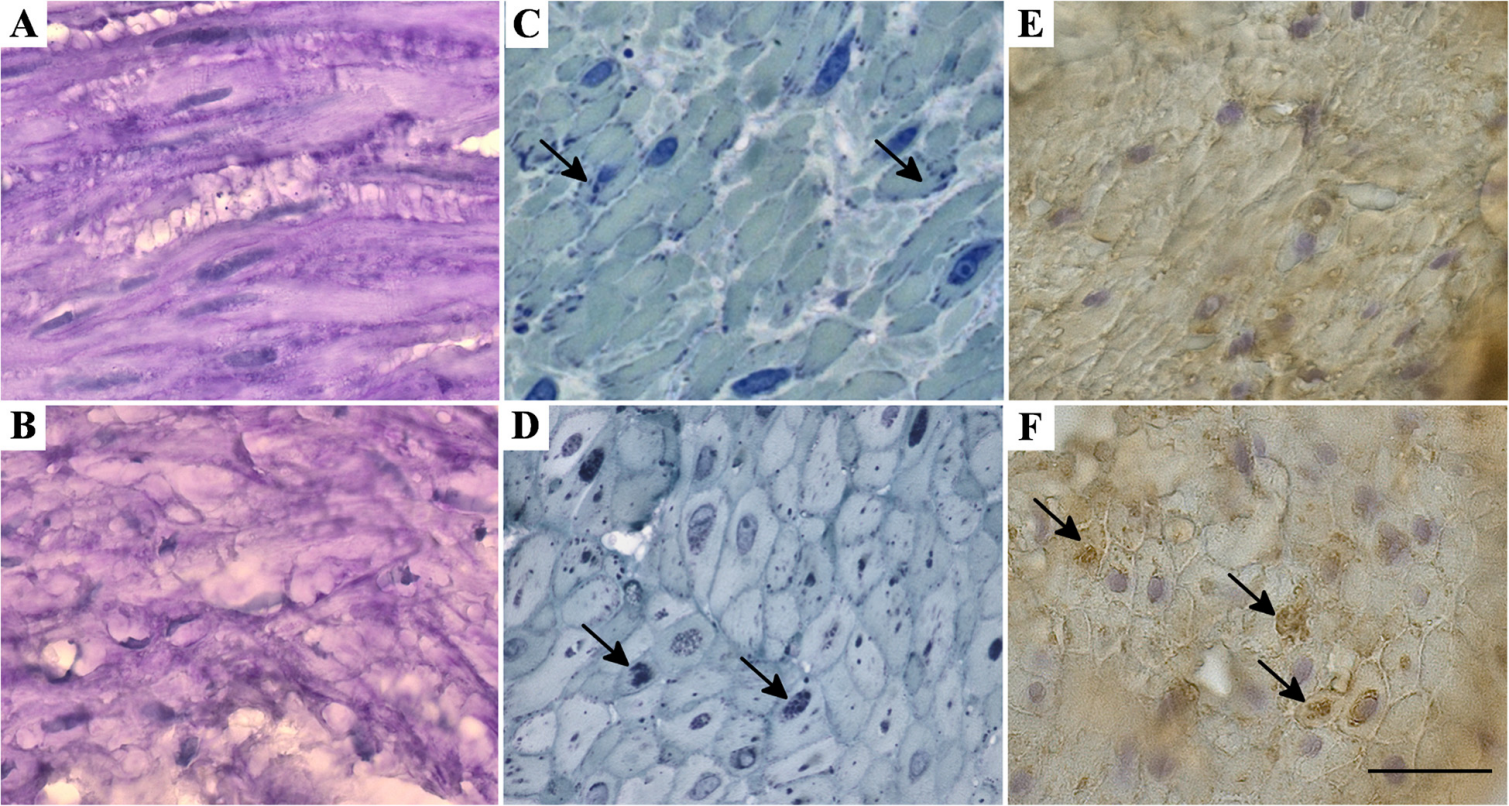
**PAS staining, p62 immunohystochemistry and Toluidine blue staining of arrector pili muscles from control and Pompe patients skin biopsies. A-B**: The different types of glycogen accumulation (granular glycogen versus membrane bound form) are indistinguishable with PAS staining of controls (A) and patients (B). **C-D**: Toluidine blue staining of semithin sections shows granular glycogen accumulations in control (arrows, C) and glycogenosomes (arrows, D) in Pompe patient biopsies. **E-F**: Immunohistochemistry against p62 (a marker for autophagy) shows accumulations in patient arrector pili muscle (F, arrows) but not in healthy controls (E). Scale bar for all parts: 20 μm.

Light microscopy of PAS stains did not reveal any differences in arrector pili smooth muscles from controls and Pompe patients (Figure 3A-B), which could result from the inadequacy of our tissue preparation for the proper visualization of glycogen accumulation. The toluidine blue staining of the semithin sections showed the membrane bound nature of the glycogen deposits and accumulations of autophagic vacuoles in Pompe disease cases (Figure 3C-D). The autophagic origin of these vacuoles was confirmed by p62 staining (Figure 3E-F). The role of p62 is to link the ubiquitinated cargos to the autophagy machinery for autophagic degradation; p62 is widely used as a marker for accumulation of autophagic vacuoles [16]. However, unequivocal differences could only be demonstrated by electron microscopic examination. Arrector pili smooth muscle cells from Pompe disease patients showed vastly increased glycogen storage in glycogenosomes associated with prominent, pleomorphic, mostly granular accumulations in autophagic vacuoles (Figure 2F-I). In contrast, control smooth muscle cells also showed glycogen accumulations but these were less pronounced, not as densely packed and not membrane-bound. Such granular glycogen deposits were in many cases loosely associated with sub-cellular structures, such as mitochondria or plasma membrane and endoplasmic reticulum, but were not associated with prominent autophagic vacuoles (Figure 2D-E).

### Quantification of glycogen deposition

Quantitative analysis confirmed that although many smooth muscle cells (78%) in controls contained glycogen, the typical form of glycogen was as small granular deposits. Only 7% of cells showed an elevated number of deposits and less than 1% contained membrane-bound glycogen. No glycogen deposits could be found in 22% of control cells. In contrast, 37% of Pompe disease patient-derived arrector pili smooth muscle cells contained abnormal forms of glycogen; among these 11% showed accumulation of autophagic vacuoles filling a significant part of the cytoplasm. The distribution of these different forms of glycogen deposits was significantly different between patients and controls (p > 0,0001) (Figure 1A).

We also compared the pre- and post-ERT biopsies in two cases. In the pooled quantification data of these two cases we saw a decrease in the number of cells containing glycogenosomes (33.1% vs. 29.7%) and also in those severely affected by autophagy (from 9.0% to 5.7%). The distribution for the two treated patient can be seen in Table 2. Due to the small number of subjects (n = 2) and the high individual variation in patients, a comprehensive statistical analysis of the patterns of intracellular glycogen deposition before and after ERT was not possible, even though the decrease in cells affected by autophagy compared to all other cells was significant (p = 0.003) (Figure 1B).

**Table 2:** Distribution of glycogen content in arrector pili smooth muscle cells in patients prior to and during ERT

	**1**	**2**	**3**	**4**	**5**
**P4 before ERT**	15, 6	31, 8	2, 6	41, 1	8, 9
**P4 after ERT**	21, 0	42, 0	1, 4	29, 7	5, 8
**P5 before ERT**	9, 1	55, 3	1, 5	25, 0	9, 1
**P5 after ERT**	16, 7	44, 0	4, 2	29, 6	5, 6

Categories are as described in the legends of Figure 1. The numbers in the table represent percentages of the total counted cell numbers.

## Discussion

In the present study we describe the prominent glycogen accumulation in the arrector pili smooth muscle fibers as an easily accessible representative of smooth muscle in adult patients with Pompe disease. The electron microscopic finding of glycogenosomes associated with autophagic vacuoles distinguished adult Pompe disease patients from controls. Glycogenosomes, i.e. glycogen-filled lysosomes are the hallmark of Pompe disease [1-3,5,7]. Light microscopy of PAS stained sections of formalin fixed material was, by itself, not sufficient to differentiate samples from Pompe disease patients and controls. The analysis of follow-up samples from patient undergoing ERT suggested a mild beneficial therapeutic effect, as indicated by a slight reduction in the number of glycogenosomes and autophagic vacuoles in the arrector pili muscles. This effect needs to be verified in a larger group of patients.

In addition, it was shown that patients display a correlation between autophagy and skeletal muscle atrophy, and disease progression. In a single adult case, ERT resulted in a decrease of autophagic aggregates and skeletal muscle fibre vacuolisation [17]. A similar trend was seen in our *de novo* treated patients. The presence of excessive autophagy in smooth muscle associated with abnormal glycogen accumulation is particularly interesting since autophagy is thought to have a direct role in the pathomechanism of Pompe disease [18,19] and its negative effect to ERT efficiency has been demonstrated in animal models [20]. Because the morphological alterations of skeletal and smooth muscle are remarkably similar, the arrector pili muscle appears to be suitable for the study of these pathomechanisms in human patients.

In conclusion, our findings suggest that the standardized skin biopsy technique could offer an easy and minimally invasive way to screen for smooth muscle involvement in Pompe disease and warrant further studies in larger cohorts of patients. Furthermore, arrector pili muscle pathology might even be useful as a surrogate marker for the involvement of smooth muscles in other tissues, reflecting disease severity.

## Abbreviations

ERT: Enzyme replacement therapy; GAA: Acid alpha-glucosidase; H&E: Hematoxylin and eosin; PAS: Periodic acid Schiff stain.

## Competing interests

F. Hanisch received lecturer honoraria and travel fees from Genzyme, Astellas, and Biomarin Inc. I. Katona and J. Weis have no competing interests.

## Authors’ contributions

The clinical examination of the patients was performed by FH. The experimental work, including immunohistochemistry, light microscopy, electron microscopy, quantification of glycogen content of the cells and statistical analysis was done by IK. The manuscript was written by IK, JW and FH. All authors read and approved the final manuscript.
